# Higher satisfaction with an alternative collection device for stool sampling in colorectal cancer screening with fecal immunochemical test: a cross-sectional study

**DOI:** 10.1186/s12885-018-4290-0

**Published:** 2018-04-02

**Authors:** Hye Young Shin, Mina Suh, Kui Son Choi, Sang-Hyun Hwang, Jae Kwan Jun, Dong Soo Han, You Kyoung Lee, Jae Hwan Oh, Chan Wha Lee, Do-Hoon Lee

**Affiliations:** 1 National Cancer Control Institute, National Cancer Center, 323 Ilsan-ro, Ilsandong-gu, Goyang, 10408 Republic of Korea; 2 Graduate School of Cancer Science and Policy, National Cancer Center, Goyang, 10408 Republic of Korea; 3 Department of Laboratory Medicine, University of Ulsan College of Medicine and Asan Medical Center, Seoul, 05505 Republic of Korea; 4 Department of Gastroenterology, Hanyang University Guri Hospital, 153, Gyeongchun-ro, Guri, 11923 Republic of Korea; 5 Department of Laboratory Medicine and Genetics, Soonchunhyang University Bucheon Hospital and Soonchunhyang University College of Medicine, 170, Jomaru-ro, Wonmi-gu, Bucheon-si, 14584 Republic of Korea; 6 Center for Colorectal Cancer, National Cancer Center Hospital, National Cancer Center, 323 Ilsan-ro, Ilsandong-gu, Goyang, 10408 Republic of Korea; 7 Center for Cancer Prevention & Detection, National Cancer Center Hospital, National Cancer Center, 323 Ilsan-ro, Ilsandong-gu, Goyang, 10408 Republic of Korea; 8 Department of Laboratory Medicine, Center for Diagnostic Oncology, National Cancer Center Hospital, National Cancer Center, 323 Ilsan-ro, Ilsandong-gu, Goyang, 10408 Republic of Korea; 9 College of Nursing, Korea University, Seoul, South Korea

**Keywords:** Clorectal neoplasm, Fecal occult blood test, Colorectal cancer screening, Satisfaction

## Abstract

**Background:**

Identifying preferences for stool collection devices may help increase uptake rates for colorectal cancer screening via fecal immunochemical test (FIT). This study surveyed satisfaction with different devices utilized to collect stool samples for FIT: a conventional container and a sampling bottle (Eiken OC-Sensor).

**Methods:**

This cross-sectional study was conducted at the National Cancer Center, Korea. Participants aged 50–74 years who used either a conventional container or a sampling bottle to collect a stool sample for FIT were asked to complete a questionnaire designed to survey their satisfaction with the stool collection process and their intentions to undergo FIT in subsequent screening rounds. In total, 1657 participants (1224 conventional container, 433 sampling bottle) were included for analysis.

**Results:**

Satisfaction with the sampling bottle was higher than that with the conventional container (79.9% vs.73.0%, *p* = 0.005, respectively; aOR = 1.52, 95% CI: 1.16–2.00). Participants satisfied with the sampling bottle were more likely to be female, be of younger age (50–64 years old), have higher household income, and have prior experience with FIT. Intentions to undergo subsequent screening were stronger among those given the sampling bottle than those given the conventional container (aOR = 1.78, 95% CI: 1.28–2 .48).

**Conclusions:**

Satisfaction with the stool collection process was higher with the sampling bottle. However, additional studies are needed to validate whether the increased satisfaction and stronger intentions to undergo subsequent screening with the sampling bottle could actually lead to increased uptake in subsequent rounds, along with analysis of the device’s cost effectiveness.

**Electronic supplementary material:**

The online version of this article (10.1186/s12885-018-4290-0) contains supplementary material, which is available to authorized users.

## Background

Colorectal cancer (CRC) is the third most common cancer in men and the second most common cancer in women worldwide. While CRC has remained more prevalent in Western developed countries, increases in CRC have recently been noted in Asian countries [1, 2]. In Korea, regardless of sex, the incidence of CRC has steeply increased over the past ten years, more so than other cancers. In 1999, the incidence of CRC in Korean men was the fourth highest among all cancers; by 2014, it ranked third [3].

A stool-blood test is a non-invasive, safe, and simple test with which to detect CRC, and has been found to help reduce the incidence and mortality thereof [4–6]. Accordingly, several countries have adopted stool-blood tests for CRC screening as part of nationwide screening programs [2, 7]. In Korea, the National Cancer Screening Program (NCSP) began to offer annual CRC screening with stool-blood testing to individuals aged 50 years and older in 2004: the type of stool-blood test offered is a fecal immunochemical test (FIT) that requires only a single stool sample [8], which is relatively convenient, as other stool-blood tests necessitate diet restrictions and several stool samples [9, 10]. However, the uptake rate for CRC screening in 2012 was as low as 25.0% [8], which is lower than rates in other countries offering organized CRC screening [11–13].

In Korea, non-attendance to CRC screening has been found to be affected by the stool collection process [14]. Individuals targeted for CRC screening visit a clinic where they are provided with an approximately 20-cm^3^, plain, plastic container (referred to as a conventional container in this study) in which to deposit their stool sample for FIT. The conventional container comprises a short sampling probe (2.0 cm) and a simple cap. Individuals undergoing FIT must pay close attention when collecting their stool sample, and most people wrap the device to prevent odors from escaping. While these barriers have been found to be sources of inconvenience, and even distress, to would-be participants, overcoming them could help increase uptake rates for CRC screening [15, 16]. Accordingly, identifying preferences for stool collection devices would be important thereto.

In the present study, we aimed to compare participants’ satisfaction with a conventional container and an alternative sampling bottle. Also, we sought to identify intentions to undergo FIT in subsequent screening rounds according to preferences for each of these devices.

## Methods

### Study setting and subjects

Between February 2013 and August 2014, this cross-sectional study was conducted at the National Cancer Center (NCC) in Goyang City, Republic of Korea. The target population of the study was restricted to individuals aged 50–74 years with no history of cancer. Study participants, residents in Goyang City, were recruited by both invitation letters and in collaboration with the Center for Cancer Prevention and Detection at the NCC. We initially mailed invitation letters to the entire target population for CRC screening among residents in Goyang City. Those who contacted the study researchers received either a sampling bottle or a conventional container randomly after providing verbal consent for the study via phone interviews. However, the enrolled number of study participants was small at 103. Thus, we sought to collaborate with the Center for Cancer Prevention and Detection at the NCC. As a collaborative effort, phone interviews were performed to recruit individuals who were already scheduled to undergo FIT screening as part of the NCSP at the center. That is, participants who had not obtained a conventional container, which is currently used in the NCSP, were given either a sampling bottle or a conventional container randomly. At the same time, in addition to this recruitment, we collected participants at the center (or clinic) directly: These individuals had already been given a conventional container and were visiting the clinic to submit their stool specimens. Thus, they were included in the study group given the conventional container. Finally, a total of 1657 participants who provided written informed consent to participate in the study upon submitting their stool specimen at the clinic were included in this study, and a cross-sectional analysis comparing differences in satisfaction between the stool collection devices was conducted. This study was approved by the Institutional Review Board of the National Cancer Center, Korea (NCCNCS-12-683).

### Data collection and outcome measures

All participants utilized the given stool collection devices for stool sampling at home. The stool collection devices have been illustrated elsewhere [17]. The conventional container comprised a plain pot with a 2.0-cm-long sampling probe and an attached cap. The sampling bottle consisted of a small tube comprising a thin and long sampling probe (4.2 cm long) with a grooved, spiraling tip and a twistable structure to open the cap (OC-Sensor; Eiken Chemical Co., Ltd., Tokyo, Japan) [18]. For FIT analysis in the laboratory, stool specimens collected via the conventional container at home were resampled using the sampling bottle and placed in the analyzer machine, while those collected via the sampling bottle were left in the sampling bottle and placed directly in the machine for FIT analysis. Those who visited to the Center for Cancer Prevention and Detection at the NCC to submit their samples were asked to complete a questionnaire designed to survey their satisfaction with the stool collection process.

The survey questionnaire was developed and modified from that reported in previous studies [16, 19]. To ensure the validity of the contents of the questionnaire, specialists in gastroenterology, epidemiology, and public health modified the questionnaire (Additional file 1). Thereafter, to investigate the readability of the questionnaire, a pretest was conducted with 35 individuals of ages older than 50 years old who were employed by the National Cancer Center. Data regarding satisfaction with the stool collection process were collected via the questionnaire for two dimensions: (1) satisfaction with each phase, including putting the stool specimen into a device, odor from the device after obtaining the stool sampling, and submission of the device at the clinic, and (2) overall satisfaction with the entire process, from stool sampling at home to submission of the stool specimen at the clinic. All satisfaction-related items were surveyed with the following question, “Did you feel satisfied with the stool collection process?” except for the item regarding odor, which asked “Did you notice odor coming out of the device after you stored the stool specimen in the device?” Responses were measured on a five-point Likert scale (1 = strongly agree, 2 = agree, 3 = uncertain, 4 = disagree, 5 = strongly disagree). We defined satisfaction as a Likert score of 1–2 and dissatisfaction as a Likert score of 3–5, except in regards to odor, wherein satisfaction was defined as a Likert score of 4–5 and dissatisfaction as a Likert score of 1–3.

Data on intentions to undergo FIT in subsequent screening rounds were collected via a single item: “Would you be willing to undergo stool sampling with the same device next year?” Responses were measured on a five-point Likert scale (1 = strongly agree, 2 = agree, 3 = uncertain, 4 = disagree, 5 = strongly disagree). We defined intentions to undergo FIT as a Likert score of 1–2 and no intention as a Likert score of 3–4. Additionally, we examined demographic factors, including sex, age, monthly household income, level of education, having supplemental cancer insurance, adherence to regular medical check-ups, self-rated health status, and experiences with FIT in the past.

## Statistical analysis

The chi-square test was conducted to evaluate the participants’ general characteristics and the intervention effect unadjusted. Logistic regression was also applied to estimate the odds ratios (OR) for satisfaction with sample collection and intentions to undergo FIT in subsequent screening rounds according to the stool collection device, after adjusting for covariates. All statistical analyses were conducted with SAS statistical software (version 9.3; SAS Institute, Inc., Cary, North Carolina, USA), and all *P*-values < 0.05 were considered statistically significant.

## Results

A total of 1657 participants (1224 conventional container, 433 sampling bottle) were included for analysis. The majority thereof was female, 50–64 years old, and had prior experience with FIT. Differences between the study groups in regards to education year (*p* = 0.030) and FIT experience in the past (*p* < 0.001) were significant, while those for other factors were not statistically significant (Table 1).

**Table 1:** General characteristics of the study groups

Characteristics	Total	Conventional container	Sampling bottle	*p*-value
		*n* (%)	*n* (%)	
Overall	1657 (100.0)	1224 (73.9)	433 (26.1)	
Gender				
Male	681 (41.1)	518 (42.3)	163 (37.6)	0.089
Female	976 (58.9)	706 (57.7)	270 (62.4)	
Age group, years				
50–64	1176 (71.0)	860 (70.3)	316 (73.0)	0.284
65–74	481 (29.0)	364 (29.7)	117 (27.0)	
Monthly household income, US$				
≤ 1999	525 (31.7)	380 (31.1)	145 (33.5)	0.512
2000–3999	566 (34.2)	427 (34.9)	139 (32.1)	
≥ 4000	566 (34.2)	417 (34.1)	149 (34.4)	
Education, years				
≤ 9	447 (27.0)	314 (25.7)	133 (30.7)	0.030
10–12	654 (39.5)	479 (39.1)	175 (40.4)	
≥ 13	556 (33.6)	431 (35.2)	125 (28.9)	
Supplemental insurance for cancer				
No	576 (34.8)	422 (34.5)	154 (35.6)	0.683
Yes	1081 (65.2)	802 (65.5)	279 (64.4)	
Regular medical check-up				
No	1374 (82.9)	1023 (83.6)	351 (81.1)	0.232
Yes	283 (17.1)	201 (16.4)	82 (18.9)	
Self-rated health status				
Good	557 (33.6)	431 (35.2)	126 (29.1)	0.056
Fair	824 (49.7)	598 (48.9)	226 (52.2)	
Bad	276 (16.7)	195 (15.9)	81 (18.7)	
History of FIT				
No	403 (24.3)	240 (19.6)	163 (37.6)	<.0001
Yes	1254 (75.7)	984 (80.4)	270 (62.4)	

*FIT* fecal immunochemical test

**Fig. 1:**
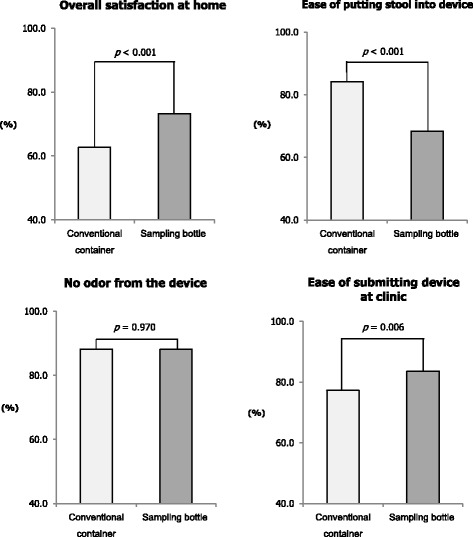
Satisfaction with the stool collection process for FIT

Figure 1 depicts the participants’ satisfaction with the process of stool collection for FIT. Participants who were given a sampling bottle reported significantly greater satisfaction in regards to the entire stool collection process (*p* < 0.001) and ease of submitting the device at a clinic (*p* = 0.006). Participants who were given a conventional container showed significantly greater satisfaction with putting the stool specimen into the device (*p* < 0.001). Experiences of odor escaping the devices showed no statistically significant difference between participants given a conventional container and those given a sampling bottle (*p* = 0.970).

Compared to satisfaction with the conventional container, that with the sampling bottle was higher (79.9% vs.73.0%, *p* = 0.005, respectively) (Table 2). After adjusting for covariates, overall satisfaction with the collection process was significantly higher in the sampling bottle group, compared to the conventional container group (aOR = 1.52, 95% CI: 1.16–2.00). Participants satisfied with the sampling bottle were more likely to be female, be of ages 50–64 years old, have a monthly household income of more than $4000, have 13 or more education years, have supplemental cancer insurance, and have experience with FIT in the past (Table 2). Intentions to undergo FIT in subsequent screening rounds were significantly greater in the sampling bottle group than the conventional container group (aOR = 1.78, 95% CI: 1.28–2.48) (Table 3).

**Table 2:** Participant satisfaction according to stool collection device

Characteristics	Satisfaction (Yes), n (%)			Sampling bottle vs. Conventional container	
	Sampling bottle	Conventional container	*p*-value*	_C_OR (95% CI)	aOR (95% CI)
	(*n* = 433)	(*n* = 1224)			
Overall	346 (79.9)	894 (73.0)	0.005	1.47 (1.12–1.92)	1.52 (1.16–2.00)
Sex					
Male	139 (79.8)	382 (73.8)	0.121	1.40 (0.91–2.15)	1.55 (0.99–2.43)
Female	216 (80.0)	512 (72.5)	0.016	1.52 (1.08–2.13)	1.53 (1.08–2.16)
Age group, years					
50–64	259 (82.0)	606 (70.5)	<.001	1.91 (1.38–2.63)	1.96 (1.41–2.72)
65–74	87 (74.4)	288 (79.1)	0.280	0.77 (0.47–1.24)	0.81 (0.48–1.36)
Monthly household income, US$					
≤ 1999	115 (79.3)	293 (77.1)	0.587	1.14 (0.71–1.82)	1.27 (0.77–2.08)
2000–3999	109 (78.4)	324 (75.9)	0.540	1.16 (0.73–1.83)	1.27 (0.79–2.05)
≥ 4000	122 (81.9)	277 (66.4)	0.000	2.28 (1.44–3.63)	2.26 (1.41–3.63)
Education, years					
≤ 9	107 (80.5)	240 (76.4)	0.351	1.27 (0.77–2.10)	1.40 (0.83–2.35)
10–12	137 (78.3)	347 (72.4)	0.132	1.37 (0.91–2.07)	1.42 (0.93–2.16)
≥ 13	102 (81.6)	307 (71.2)	0.021	1.79 (1.09–2.95)	1.82 (1.08–3.06)
Supplemental insurance for cancer					
No	126 (81.8)	326 (77.3)	0.238	1.33 (0.83–2.12)	1.48 (0.90–2.44)
Yes	220 (78.9)	568 (70.8)	0.009	1.54 (1.11–2.13)	1.57 (1.12–2.18)
Regular medical check-up					
No	277 (78.9)	749 (73.2)	0.034	1.37 (1.02–1.83)	1.40 (1.04–1.89)
Yes	69 (84.2)	145 (72.1)	0.033	2.05 (1.05–4.00)	2.78 (1.35–5.75)
Self-rated health status					
Good	109 (86.5)	313 (72.6)	0.001	2.42 (1.39–4.20)	2.61 (1.48–4.59)
Fair	172 (76.1)	446 (74.6)	0.652	1.09 (0.76–1.55)	1.12 (0.78–1.63)
Bad	65 (80.3)	135 (69.2)	0.062	1.81 (0.97–3.38)	1.93 (1.01–3.71)
History of FIT					
No	128 (78.5)	167 (69.6)	0.047	1.60 (1.01–2.54)	1.58 (0.99–2.53)
Yes	218 (80.7)	727 (73.9)	0.021	1.48 (1.06–2.07)	1.50 (1.07–2.10)

_*C*_*OR* crude odds ratio, *aOR* adjusted odds ratio, *CI* confidence interval, *FIT* fecal immunochemical test

^*^p-values were given from chi-square test

**Table 3:** Intention to be rescreened via FIT in subsequent screening rounds

Characteristics	Satisfaction (Yes), *n* (%)			Sampling bottle vs. Conventional container	
	Sampling bottle	Conventional container	*p*-value*	_C_OR (95% CI)	aOR (95% CI)
	(*n* = 433)	(*n* = 1224)			
Overall	381 (88.0)	1000 (81.7)	0.003	1.64 (1.19–2.27)	1.78 (1.28–2.48)
Sex					
Male	143 (87.7)	427 (82.4)	0.110	1.52 (0.91–2.56)	1.86 (1.08–3.21)
Female	238 (88.2)	573 (81.2)	0.009	1.73 (1.14–2.61)	1.76 (1.15–2.67)
Age group, years					
50–64	279 (88.3)	696 (80.9)	0.003	1.78 (1.21–2.61)	1.88 (1.27–2.78)
65–74	102 (87.2)	304 (83.5)	0.342	1.34 (0.73–2.47)	1.52 (0.80–2.88)
Monthly household income, US$					
≤ 1999	126 (86.9)	310 (81.6)	0.147	1.50 (0.87–2.59)	1.63 (0.92–2.90)
2000–3999	120 (86.3)	359 (84.1)	0.522	1.20 (0.69–2.07)	1.33 (0.76–2.34)
≥ 4000	135 (90.6)	331 (79.4)	0.002	2.51 (1.38–4.56)	2.61 (1.42–4.82)
Education, years					
≤ 9	115 (86.5)	257 (81.9)	0.232	1.42 (0.80–2.52)	1.47 (0.82–2.65)
10–12	159 (90.9)	390 (81.4)	0.004	2.27 (1.29–3.98)	2.66 (1.49–4.74)
≥ 13	107 (85.6)	353 (81.9)	0.336	1.31 (0.75–2.29)	1.35 (0.76–2.41)
Supplemental insurance for cancer					
No	140 (90.9)	343 (81.3)	0.005	2.30 (1.26–4.20)	2.73 (1.45–5.15)
Yes	241 (86.4)	657 (81.9)	0.087	1.40 (0.95–2.06)	1.46 (0.99–2.16)
Regular medical check-up					
No	306 (87.2)	839 (82.0)	0.025	1.49 (1.05–2.12)	1.59 (1.11–2.28)
Yes	75 (91.5)	161 (80.1)	0.020	2.66 (1.14–6.22)	3.49 (1.42–8.54)
Self-rated health status					
Good	112 (88.9)	363 (84.2)	0.194	1.50 (0.81–2.77)	1.65 (0.88–3.10)
Fair	199 (88.1)	480 (80.3)	0.009	1.81 (1.16–2.84)	1.94 (1.22–3.09)
Bad	70 (86.4)	157	0.242	1.54 (0.74–3.19)	1.67 (0.79–3.53)
History of FIT					
No	137 (84.1)	189	0.184	1.42 (0.84–2.39)	1.41 (0.83–2.39)
Yes	244 (90.4)	811	0.002	2.00 (1.29–3.10)	2.05 (1.32–3.19)

_*C*_*OR* crude odds ratio, *aOR* adjusted odds ratio, *CI* confidence interval, *FIT* fecal immunochemical test

^*^p-values were given from chi-square test

## Discussion

The nature of the stool collection process for stool-blood test can be psychologically distressing, eliciting disgust, embarrassment, and unpleasantness [10, 20, 21]. Increasing satisfaction with the stool collection process might help as an alternative strategy to improve uptake rates of CRC screening [21]. In the current study, we evaluated satisfaction with the stool collection process according to different stool collection devices (conventional container vs. sampling bottle). Higher satisfaction with the stool collection process was reported by participants given a sampling bottle than those given a conventional container. Accordingly, those who used the sampling bottle were more likely to have intentions to undergo FIT in subsequent screening rounds, which could be related to increased satisfaction with the stool collection process.

Compared with other stool collection devices, the conventional container (similar to a fecal specimen pot) has been found to garner less satisfaction [16]. Conventional containers have a short sampling probe that places the hand close to the operator’s feces, and the container has no indicator as to how much sample has been collected, causing potential confusion. Comparatively, sampling bottles have a relatively longer sampling probe with which to collect stool specimens, and only a small amount (~ 10 mg) of sample needs to be placed in the bottle through a filter [18], which could ease barriers to and increase the attractiveness of FIT. Also, the smaller and more discreet sampling bottle, compared to the conventional container, could potentially reduce embarrassment with and boost opinions on submitting the collected stool specimen at a clinic, which might explain our finding of particularly higher satisfaction with specimen submission using the sampling bottle.

In the present study, favorable attitudes toward satisfaction with and intentions to undergo FIT in the next screening round were prominent among women (both men and women for intentions), younger participants aged 50–64 years, and individuals with greater income who were given a sampling bottle. Similar to the uptake rate for FIT of other countries, rates in Korea are higher in women [8], but the overall uptake rate in the target population is quite low at 25.0% (women 25.8, men 24.1%). Thus, our findings of higher satisfaction in women and stronger intentions to undergo subsequent screening in both sexes suggest that the use of a sampling bottle might be helpful in increasing the uptake rate, although our findings were not directly representative of actual uptake rates for CRC screening. Similarly, higher satisfaction in the younger group, where the compliance is commonly low [8, 22], suggests that there is potentially greater room for improvement in that groups. In addition, as participants of higher income status reported greater satisfaction and stronger intentions to undergo subsequent screening with FIT than their low income counterparts, other strategies that facilitate mailing of stool specimens might be needed to increase the rates among lower income participants, although higher income participants would benefit as well. Moreover, participants who had previously undergone FIT with the conventional container expressed higher satisfaction with the sampling bottle. Their perceptions of the sampling bottle might be more reliable, since they had previously used a conventional container in the NCSP system. Thus, they may be better suited to compare their experiences with both devices.

Despite these positive findings, the small opening of the sampling bottle led to significantly less satisfaction with putting the stool specimen into the device. This was particularly apparent among older adults who have physical difficulties with handling the stool specimen. Also, with regard to odor coming out from the devices, we noted no difference between the sampling bottle and conventional container, although the sampling bottle has a more sophisticated tube cap. This finding is probably associated with usual practices: those who used the conventional container wrapped the device several times in wrapping materials or a zip-lock bag to prevent unwanted odors. Finally, our findings suggest that an alternative device that compensates for these disadvantages should be developed, and education on the stool collection process, including the use of the device, should be repeatedly conducted in detail considering the age of the target population for CRC screening.

Our study has several limitations. First, as stated above, the opinions of individuals who were screened for the first time with FIT likely add limited value in regards to the comparison of the stool collection devices. That said, higher satisfaction and intentions to be rescreened were more likely to be reported among participants who already had experiences with FIT, which suggests that our inclusion of the first-time screeners might have had little effect on our findings. However, our direct recruitment at the clinic might have led to a predominance of regular screeners who may not accurately reflect the attitudes of first-time screeners. Second, we evaluated intentions to undergo FIT instead of actual uptake rates of subsequent screening rounds. However, intentions could be a predictor of completion of CRC screening [23]. Finally, the number of participants who used a sampling bottle was small, compared to those who used a conventional container. This aspect reflects one limitation of the cross-sectional study design and the location of participant recruitment, a clinic for FIT. That is, most study participants were already scheduled to undergo CRC screening with FIT, and stool collection devices were distributed to them based on whether they had already received a conventional device or not.

Despite these limitations, to our knowledge, the current study is the first to compare satisfaction with FIT according to type of stool collection device. Previous studies that attempted to evaluate different sampling devices could not make direct comparisons in regards to the effectiveness of the devices, because they included number of stools, number of samples per stool, and different sampling methods (guaiac fecal occult test versus FIT) with different sampling devices [10, 15, 16, 21]. Also, the present study identified satisfaction in regards to individual phases of the stool collection process according to the devices, which allowed us to provide insights into opportunities for developing a more user-friendly device.

## Conclusions

In conclusion, the majority of the participants in the present study showed higher satisfaction and stronger intentions to undergo subsequent screening rounds with the use of a sampling bottle. These findings might contribute to establishing a strategy with which to increase CRC screening by overcoming barriers to FIT. However, additional studies are needed to validate whether the noted increases in satisfaction and greater intentions to undergo subsequent screening with the sampling bottle could actually lead to increased uptake in subsequent rounds. Furthermore, before implementing the sampling botte in the NCSP, evaluation of the cost effectiveness of each collection device in relation to improved CRC screening rates is needed to justify the switch.

## Additional file

Additional file 1: FOBT Questionnaire. Questionnaire about satisfaction with the use of stool collection devices for stool sampling (fecal immunochemical test, FIT) in colorectal cancer screening. (DOCX 16 kb)

## Abbreviations

CRC: Colorectal cancer
FIT: Fecal immunochemical test
NCC: National Cancer Center
NCSP: National Cancer Screening Program
