# Supplementary material for: Higher satisfaction with an alternative collection device for stool sampling in colorectal cancer screening with fecal immunochemical test: a cross-sectional study
Source: BMC Cancer. 2018 Apr 2;18:365. doi: 10.1186/s12885-018-4290-0 (PMC5879644; doi:10.1186/s12885-018-4290-0)
Supplement: Supplementary file 1 — FOBT Questionnaire. Questionnaire about satisfaction with the use of stool collection devices for stool sampling (fecal immunochemical test, FIT) in colorectal cancer screening. (DOCX 16 kb) [file 12885_2018_4290_MOESM1_ESM.docx]

**Questionnaire about satisfaction with the use of stool collection devices for stool sampling (fecal immunochemical test, FIT) in colorectal cancer screening**

1. The following questions are related to your experiences with the stool collection device during the sampling process:

|  | strongly agree | agree | uncertain | disagree | strongly disagree |
| --- | --- | --- | --- | --- | --- |
| (1) Did you feel satisfied (at ease) with putting stool into the device? | ① | ② | ③ | ④ | ⑤ |
| (2) Did you feel satisfied (at ease) with submitting the device containing stool at the clinic? | ① | ② | ③ | ④ | ⑤ |
| (3) Did you notice odor coming out of the device after you stored the stool specimen in the device? | ① | ② | ③ | ④ | ⑤ |
| (4）Did you feel overall satisfaction with the stool collection process? | ① | ② | ③ | ④ | ⑤ |
| (5) Would you be willing to undergo stool sampling (FIT) with the same device next year? | ① | ② | ③ | ④ | ⑤ |

2. Do you regularly receive a comprehensive medical check-up?

① Yes ② No

3. Are you covered by private supplemental insurance for cancer?

① Yes ② No

4. What is your final academic background?

① Uneducated ② Primary school ③ Middle school

④ High school ⑤ College ⑥ Above college

5. What is the total average monthly income for all family members in your household?

① No income

② Less than ￦1,000,000 KRW

③ ￦1,000,000 - ￦1,990,000 KRW

④ ￦2,000,000 - ￦2,990,000 KRW

⑤ ￦3,000,000 - ￦3,990,000 KRW

⑥ ￦4,000,000 - ￦4,990,000 KRW

⑦ ￦5,000,000 - ￦6,990,000 KRW

⑧ ￦7,000,000 - ￦9,990,000 KRW

⑨ More than ￦10,000,000 KRW

6. How do you feel about your overall health status?

① Fairly good ② Good ③ Normal ④ Bad ⑤ Fairly bad

7. Have you ever previously performed stool sampling for colorectal cancer screening?

① Yes ② No
